# Supplementary material for: Prevalence of Violence against Providers in Heart and Lung Transplant Programs
Source: Int J Environ Res Public Health. 2023 Mar 9;20(6):4805. doi: 10.3390/ijerph20064805 (PMC10049342; doi:10.3390/ijerph20064805)
Supplement: Supplementary file 1 [file ijerph-20-04805-s001.zip › ijerph-2217218-supplementary.pdf]

Consent Form: Prevalence of Violence Against Providers in Heart and Lung Transplant Programs

Providers' Perceptions of and Experiences with Safety Threats in Heart Transplant and Lung Transplant Practices

Hello! Researchers at The Ohio State University are conducting a study to understand heart transplant and lung transplant providers' perceptions of and experiences with safety threats in their workplace, specifically related to threats from patients and their family members.

If you are interested in learning more about the study and completing our anonymous survey, you can scan this QR code [*shows provider the QR code*]. Your participation is entirely voluntary and should only take a few minutes.

Do you have any questions about this study?

Would you like to participate in this study?
